# The prevalence of obesity in mature Icelandic horses in Denmark

**DOI:** 10.1186/1751-0147-57-S1-O9

**Published:** 2015-09-25

**Authors:** Rasmus Bovbjerg Jensen, Signe Hartvig Danielsen, Anne-Helene Tauson

**Affiliations:** 1 Department of Veterinary Clinical and Animal Sciences, University of Copenhagen, Copenhagen, Denmark

## Introduction

Obesity is related to the development of several diseases like insulin resistance and laminitis in horses. The prevalence of obesity among mature Icelandic horses in Denmark has not been investigated previously.

## Objectives

This study aimed to find the prevalence of obesity among mature Icelandic horses in Denmark, to compare body condition score (BCS) based on owner perception with that of an experienced person and to correlate the BCS to body weight (BW) and morphometric measures.

## Material and methods

A total of 252 Icelandic horses (≥ 4 years; 142 geldings, 101 mares, 9 stallions) from 46 different barns were included. All horses were assigned a BCS on a scale from 1-9 (1 is poor, 5 is moderate and 9 is extremely fat) by their owner and by an experienced person. A horse with a BCS of ≥7 is considered to be obese. Two weight tapes (WT) were used to asses BW. Neck circumference and height at withers were measured, and the ratio between them calculated (NCHR).

## Results

The BCS assigned by the owners correlated with the experienced person (p=0.001, r=0.57), and horses with a BCS of 3-4, 5-6 and 7-9 were 5.9, 70.1 and 24.0 %, respectively. The BCS correlated to BW estimated with a WT (p=0.001, r=0.70) and NCHR (p=0.001, 0.34), although the latter correlation was poor. The BW was systematically 19± 15 kg higher with WT 1 than 2.

## Conclusion

The prevalence of obesity in mature Icelandic horses in Denmark was high and in agreement with previous studies in other breeds.

